# A Response to Marvin Goldfried's Article on the Immaturity of the Psy-Professions

**DOI:** 10.32872/cpe.v2i2.3105

**Published:** 2020-06-30

**Authors:** Vik Nair

**Affiliations:** a NHS Greater Glasgow and Clyde, Glasgow, United Kingdom

Marvin Goldfried’s article (Goldfried, 2020) critiqued the lack of consensus within the psy-professions, articulating reasons for this, but without mentioning *power* or *interest*. I believe the preoccupation with new theories described by Goldfried arises from our inability to discard unworkable ideas, despite ample empirical or conceptual grounds for doing so, because of the workings of power.

If *technology* is the ability to understand and manipulate the non-human material world, *social power* is the ability to influence the behaviour of other humans. Social power varies according to the identities of the parties involved, while technology does not. Disciplines concerned with humans affect and are affected by social power, immediately creating uncertainty. People do not passively accept the effects of new knowledge, but seek to achieve outcomes favourable to their interests by taking control over that knowledge (“History is written by the victors”). Research findings are not determined solely by empirical data, but also through the exercise of ideological power. Social dynamics affect what is asked, what answers are acceptable, how data is interpreted and how much attention is paid to conclusions.

Our profession's immaturity is not for want of empirical data. Questions that may have been answerable decades ago persist because the answers have been unacceptable to powerful interests. Would we really expect the psychometric industry (enmeshed with clinical psychology because of the power it affords the profession) to accept that there is no evidence to support the existence of *g* or temporally and contextually stable personality? Would the legal systems in our societies suddenly shift from punishment to pragmatism, as proposed by Skinner (1973) almost half a century ago, simply because of empirical data refuting free will? Is the persistence of psychiatric diagnosis due to its utility or to the power of the psychiatric profession? How many expert witnesses in legal cases point out to judges that data obtained through self-report psychometrics is in no way objective?

This is not to suggest that change cannot happen, but that social power is as least as important as “truth” in determining how knowledge develops. Attempts to increase the maturity of our discipline have to take account of power and interest.
